# Utility of obesity indicators for metabolically healthy obesity: an observational study using the Korean National Health and Nutrition Examination Survey (2009-2010)

**DOI:** 10.1186/1471-2458-14-1166

**Published:** 2014-11-14

**Authors:** Min-Kyu Choi, Young-Ah Han, Yong Kyun Roh

**Affiliations:** Department of Family Medicine, Kangnam Sacred Heart Hospital, College of Medicine, Hallym University, Seoul, 150-950 Korea

**Keywords:** Metabolically healthy obesity, Waist circumference, Waist height ratio, Body mass index

## Abstract

**Background:**

Obese individuals who are not at an elevated risk for cardiovascular disease are described as having metabolically healthy obesity (MHO). We sought to identify clinically useful indicators of MHO.

**Methods:**

Records of the Korean National Health and Nutrition Examination Survey (2009-2010) were used to analyze 3,770 obese subjects (body mass index ≥ 25 kg/m^2^), who were divided into metabolic syndrome and MHO groups. Persons who met less than 3 of the criteria of metabolic syndrome (MS) were defined as having MHO. We estimated age-specific prevalence rates according to the number of MS criteria that were satisfied (patients meeting 0, ≤1, and ≤2 criteria of MS). Receiver operating characteristic analysis was performed to identify the best indicators of MHO.

**Results:**

The prevalence of MHO among obese patients decreased with age. When MHO was defined by the fulfillment of ≤2 criteria of MS, the areas under the curves (AUC) for waist circumference and waist-to-height ratio were 0.743 and 0.747 in men and 0.712 and 0.741 in women, respectively. Waist circumference and waist-to-height ratio were the most accurate predictors of MHO for all investigated definitions.

**Conclusions:**

Waist circumference and waist-to-height ratio provide useful indicators for diagnosing MHO, and are more accurate than body mass index, fat percentage, or weight-adjusted appendicular skeletal muscle mass in the Korean population.

## Background

As the obese population increases worldwide, the incidence of metabolic syndrome (MS) is also rising. People who are obese have a higher risk of cardiovascular disease (CVD) and diabetes [1]. MS is associated with a higher risk of a variety of diseases and conditions, including CVD, insulin resistance, diabetes, hypertension, and hyperlipidemia [2]. A recent study reported that the prevalence of MS in Korea was between 4.6% among teenagers and 25.0% among people in their fifties [3].

Body mass index (BMI) is widely used as an indicator of obesity, but it has several limitations for assessing metabolic or health status, particularly for persons who would commonly be classified as obese [4]. Indeed, some individuals with high BMIs are not at excess risk for the diseases that are generally associated with obesity; these persons are described as having metabolically healthy obesity (MHO) [5, 6]. Persons with MHO appear to have lower levels of subcutaneous fat and lipid accumulation in the liver than persons with other types of obesity, despite similar body fat compositions. Insulin sensitivity, blood pressure, lipid profiles, and inflammation-related factors (plasma C-reactive protein) are normal in persons with MHO [7, 8]. Further, individuals with MHO generally exercise more regularly and were much less likely to smoke than individuals with other types of obesity. In addition, in an 11-year observational study, individuals with MHO did not exhibit a greater risk of CVD or diabetes than normal-weight comparators [9]. Reports on the prevalence of MHO vary according to patient characteristics and diagnostic criteria. Studying an American population, Meigs et al. found that 37.0% of obese subjects (BMI > 30 kg/m^2^) did not have metabolic syndrome (MS) or a significantly increased risk of CVD [9]. Studying an adult Korean population, Lee et al. found that 47.9% of obese subjects (BMI ≥ 25 kg/m^2^) had MHO [10].

The definition of MHO has not been clearly established thus far, but it is generally identified by blood pressure, body measurements (such as abdominal circumference), triglyceride, cholesterol, fasting blood sugar, and homeostasis model assessment [6, 11]. However, there is no single established criterion for MHO, and the incidence rate differs according to the criteria used in a previously unquantified manner.

Accordingly, in this study, we investigated how the prevalence of MHO in Korea varied when it was assessed using different criteria, which were selected from the 5 diagnostic criteria for metabolic syndrome: waist circumference, blood pressure, fasting blood sugar, triglyceride, and high-density lipoprotein cholesterol. We further assessed several indicators of obesity that may be useful for diagnosing MHO, both in Korea and worldwide: body mass BMI, waist circumference, weight-adjusted appendicular skeletal muscle mass (ASM), fat percentage, and waist-to-height ratio (WtHR).

There has been little previous research on MHO in Asia, but we expect that separate investigation is necessary because the definition of obesity varies internationally, for example being a substantially lower BMI in Korea (≥25 kg/m^2^) than in the United States (>30 kg/m^2^).

## Methods

### Study subjects

The Korean National Health and Nutrition Examination Survey (KNHANES) is a community-based, cross sectional survey that is conducted by Division of Chronic Disease Surveillance of the Korea Centers for Disease Control and Prevention. We analyzed surveys collected from 2009 to 2010, representing the third year of the KNHANES IV (2007–2009) survey and the first year of the KNHANES V (2010–2012) survey. Associated sampling and data collection procedures have been described in detail previously [12]. The January 2009 to December 2010 survey data comprised 14,633 subjects aged 20 years or older. Of these, 4,356 subjects had a BMI ≥ 25 kg/m^2^, which is the threshold for obesity set by the World Health Organization Asia Pacific guidelines [13]. We excluded participants who had missing data for dual-energy X-ray absorptiometry (DXA), metabolic, or anthropometric variables that were included in our analysis (n = 495); as well as those who had liver cirrhosis, chronic liver disease, or renal disease (n = 40); and those with malignancies (n = 51). After excluding the ineligible subjects, the total number of participants was 3,770 (1,904 were female), which were divided into MHO and MS groups and analyzed. The protocol of KNHANES IV and V was approved by the Korea Centers for Disease Control and Prevention Institutionalized Review Board. All participants in this survey provided written informed consent.

### Study methods

1. Body Measurements, Obesity Indicators, and Biochemical Analysis

Height and weight were measured using SECA 225 height rods (SECA, Hamburg, Germany) and GL-6000-20 scales (CAS, Seoul, Korea), respectively, to the nearest decimal point. BMI was calculated as weight (kg)/height^2^ (m^2^). Waist circumference was measured using a tape measure at the midpoint between the lowest costa (rib) and the iliac crest of the pelvis. WHtR was calculated as waist circumference (cm)/height (cm). The percentage of body fat (fat mass/total mass × 100) and appendicular skeletal muscle mass (ASM: the lean soft tissue masses for the arms and legs) were measured using DXA (QDR 4500A, Hologic Inc., Waltham, MA, USA) in mobile examination centers. Following previous studies, we used the body weight–adjusted appendicular skeletal muscle mass (ASM) [14].

Blood pressure was measured twice after 5 minutes of rest, and the average was used for all analyses. After 12 hours of fasting, subjects were tested for blood sugar, total cholesterol, high-density lipoprotein (HDL) cholesterol, low-density lipoprotein (LDL) cholesterol, and triglyceride levels. Based on a questionnaire that each subject had completed, he or she was interviewed one-on-one by a doctor for his/her medical history and current medications, including for high blood pressure, diabetes, and hyperlipidemia.

2) Definitions of MS and MHO

Metabolic syndrome was diagnosed if subjects met any 3 of the 5 criteria set by the American Heart Association/National Heart, Lung and Blood Institute [15]. The criteria were as follows: (1) waist circumference ≥ 90 cm for men, ≥ 80 cm for women; (2) triglyceride ≥ 150 mg/dL or taking medication for hyperlipidemia; (3) HDL cholesterol < 40 mg/dL for men, < 50 mg/dL for women; (4) blood pressure ≥ 130/85 mmHg or taking anti-hypertension medication; and (5) fasting blood sugar ≥ 100 mg/dL or taking medication for diabetes.

Three definitions of MHO were investigated: a BMI ≥ 25 kg/m^2^ and 2 or fewer of the MS criteria, a BMI ≥ 25 kg/m^2^ and at most 1 of the MS criteria, and a BMI ≥ 25 kg/m^2^ and none of the MS criteria. Subjects with MHO were compared with subjects who had a BMI ≥ 25 kg/m^2^ and met the diagnostic criteria for MS.

3) Statistical Analysis

All data on continuous and categorical variables are presented as means ± SE and proportions (SE), respectively. These summary statistics take into account the complex sampling design and KNHANES sampling weights, thereby providing nationally representative prevalence estimates. All statistical analyses were performed using SPSS Statistics, version 21.0 (SPSS Inc, Chicago, USA). The characteristics of the MHO and MS groups were compared using Student’s *t*-test for continuous variables and chi-square test for categorical variables. We used multiple logistic regression to estimate the odds ratios for MHO that were associated with various obesity indicators, adjusting for age, smoking, drinking, and physical activity. To confirm the diagnostic accuracy of each obesity indicator for MHO, we analyzed the receiver operating characteristic (ROC) for each of our three MHO definitions, and calculated the associated area under the curve (AUC). ROC analyses were used to assess the diagnostic performance of the test in terms of its sensitivity and (1-specificity), for each possible cut-off value of the test. The AUC of a diagnostic test is a summary statistic for the overall diagnostic performance of the test. AUCs are useful measures for a comparing the overall diagnostic performances of two tests. AUC results are typically categorized as uninformative (AUC = 0.5), less accurate (0.5 < AUC ≤ 0.7), moderately accurate (0.7 < AUC ≤ 0.9), or very accurate (0.9 < AUC < 1) [16]. Values of *p* < 0.05 were considered statistically significant.

## Results

### General characteristics of study subjects

The characteristics of the study subjects are presented in Table 1. Our analysis included 3,770 subjects, all of whom had a BMI ≥ 25 kg/m^2^. 56.5% of the subjects were women. In total, 2,140 subjects (56.8%) met the diagnostic criteria for MS. On the other hand, 46.7% of men and 39.9% of women met 2 or fewer of the MS criteria and, accordingly, were assigned to the MHO group. The MHO group was younger and more educated than the MS group, both before and after stratifying by gender. Men in the MHO group were more likely to exercise regularly exercise and to be non-smokers than were men in the MS group, but these differences were not evident for women (Table 1). For women specifically, MHO and MS groups had similar body weights (*p* = 0.2185) and fat percentages (*p* = 0.8175). Mean BMIs were 26.8 kg/m^2^ and 27.8 kg/m^2^ for men, and 27.2 kg/m^2^ and 28.1 kg/m^2^ for women in the MHO and MS groups, respectively.

**Table 1:** **Baseline characteristics of the study population**

	MALE			FEMALE		
	MHO	MS	p-value	MHO	MS	p-value
N	871	995		759	1145	
Education [% (SE)] high to university: ≥10	86.0 (1.3)	74.6 (1.6)	<.0001	61.6 (2.3)	36.0 (1.8)	<.0001
Income [% (SE)] lowest quartile	10.0 (1.2)	13.4 (1.3)	0.0384	16.7 (1.9)	29.9 (1.8)	<.0001
Place [% (SE)] Urban	81.2 (2.4)	79.0 (2.5)	0.2802	77.9 (3.0)	72.9 (2.9)	0.0678
Occupation [% (SE)] Yes	82.3 (1.7)	83.3 (1.3)	0.6382	54.1 (2.2)	46.6 (2.0)	0.0121
Smoking [% (SE)]			0.0066			0.5368
non-smoker	25.9 (1.7)	19.0 (1.4)		89.7 (1.4)	90.7 (1.0)	
ex-smoker	29.3 (2.0)	34.4 (1.8)		3.7 (0.8)	4.2 (0.7)	
Current	44.8 (2.0)	46.6 (1.9)		6.6 (1.3)	5.1 (0.8)	
Alcohol use [% (SE)]			0.0777			0.0003
non-drinker	12.2 (1.4)	14.6 (1.3)		29.5 (2.1)	41.3 (1.9)	
mild to moderate-drinker	68.8 (1.9)	62.6 (1.9)		67.5 (2.1)	56.2 (1.9)	
heavy-drinker	19.0 (1.6)	22.8 (1.6)		3.0 (0.7)	2.5 (0.7)	
Exercise [% (SE)] Yes	31.2 (1.8)	25.9 (1.7)	0.0265	28.6(1.9)	25.1 (1.5)	0.1166
Age	39.7 ± 0.6	47.5 ± 0.4	<.0001	44.9 ± 0.6	55.4 ± 0.6	<.0001
Weight (kg)	78.8 ± 0.3	81.0 ± 0.4	<.0001	67.0 ± 0.3	67.6 ± 0.4	0.2185
Height (cm)	171.4 ± 0.3	170.5 ± 0.3	0.0066	156.8 ± 0.3	154.9 ± 0.2	<.0001
BMI	26.8 ± 0.1	27.8 ± 0.1	<.0001	27.2 ± 0.1	28.1 ± 0.1	<.0001
Waist circumference (cm)	89.2 ± 0.3	94.4 ± 0.3	<.0001	85.6 ± 0.3	91.0 ± 0.3	<.0001
Appendicular muscle mass	31.3 ± 0.1	30.3 ± 0.1	<.0001	23.9 ± 0.1	23.6 ± 0.1	0.048
Body fat (%)	25.3 ± 0.2	26.5 ± 0.2	<.0001	37.7 ± 0.2	37.7 ± 0.2	0.8175
WHtR	0.521	0.554	<.0001	0.546	0.588	0.0007
Glucose	94.0 ± 0.6	110.5 ± 1.3	<.0001	92.4 ± 0.4	107.9 ± 1.1	<.0001
Triglyceride*	137.9 ± 3.8	242.9 ± 7.5	<.0001	95.0 ± 1.8	175.0 ± 3.7	<.0001
HDL cholesterol	49.2 ± 0.4	42.6 ± 0.4	<.0001	56.4 ± 0.4	46.8 ± 0.3	<.0001
LDL cholesterol	120.4 ± 1.3	113.3 ± 1.4	<.0001	125.1 ± 1.4	121.1 ± 1.2	0.0338
Total cholesterol	192.2 ± 1.4	196.9 ± 1.4	0.0199	195.5 ± 1.6	198.2 ± 1.4	0.2121
Systolic blood pressure	117.0 ± 0.5	125.5 ± 0.6	<.0001	113.6 ± 0.7	128.9 ± 0.6	<.0001
Diastolic blood pressure	77.8 ± 0.4	83.1 ± 0.4	<.0001	73.4 ± 0.5	79.7 ± 0.3	<.0001

*p-value after log transformation.

Continuous variables (MEAN ± SE) were compared using t-tests and categorical variables [% (SE)] were compared using chi-square tests. Regarding occupation, ‘yes’ denotes any form of employment (e.g. excluding the unemployed, homemakers, students, etc.). Regarding exercise, ‘yes’ denotes more than 30 minutes of moderate-intensity activity 3 times during a recent week.

BMI, body mass index; WHtR, waist height ratio; BP, blood pressure; MHO, metabolically healthy but obese, defined as a person who satisfies 2 or fewer criteria of metabolic syndrome; MS, metabolic syndrome.

### Associations between MHO and obesity indicators

Among men, lower values of each obesity indicator (BMI, WC, ASM, fat percentage, and WHtR) were significantly associated with MHO, as defined by the fulfillment of ≤ 2 criteria for MS (*p* trend < 0.0001). Among women, however, the ASM and FP indicators were not significantly associated with MHO (*p* trend = 0.076 and 0.8551, respectively) (Table 2). Adjusted odds ratios of ASM and fat percentage for age, smoking, drinking, and physical activity also failed to show a trend with MHO in women (*p* = 0.5331 and 0.9641, respectively), whereas all indicators were available for assessing MHO in men (Table 2).

**Table 2:** Adjusted odds ratios for metabolically healthy obesity (MHO)* according to quartiles of various obesity indicators and change in MHO percentage, using various obesity indicators divided into quartiles

	BMI		WC		ASM		FAT		WHtR	
	% (SE)	OR (95% CI)	% (SE)	OR (95% CI)	% (SE)	OR (95% CI)	% (SE)	OR (95% CI)	% (SE)	OR (95% CI)
Men										
Q1	69.1 (2.4)	ref.	79.0 (2.1)	ref.	35.1 (2.7)	ref.	61.1 (2.4)	ref.	78.4 (2.0)	ref.
Q2	54.1 (2.8)	0.77 (0.56,1.05)	59.0 (2.5)	0.71 (0.48,1.05)	51.7 (2.5)	0.99 (0.74,1.32)	49.4 (2.8)	0.90 (0.66,1.22)	56.4 (2.8)	0.56 (0.39,0.81)
Q3	48.1 (2.9)	0.43 (0.31,0.60)	32.7 (2.8)	0.43 (0.31,0.59)	53.0 (2.9)	1.09 (0.82,1.45)	50.0 (2.9)	1.10 (0.82,1.48)	36.4 (2.8)	0.28 (0.19,0.41)
Q4	33.0 (2.4)	0.39 (0.29,0.54)	28.4 (2.4)	0.13 (0.09,0.19)	62.0 (2.6)	1.28 (0.96,1.72)	43.6 (2.8)	0.96 (0.71,1.32)	23.6 (2.4)	0.12 (0.08,0.17)
p for trend	<.0001	<.0001	<.0001	<.0001	<.0001	<.0001	<.0001	<.0001	<.0001	<.0001
Women										
Q1	53.0 (2.9)	ref.	70.0 (2.4)	ref.	38.3 (2.8)	ref.	43.0 (2.7)	ref.	72.9 (2.4)	ref.
Q2	50.6 (2.7)	0.61 (0.43,0.87)	42.4 (2.7)	0.68 (0.45,1.05)	42.2 (2.7)	0.88 (0.63,1.23)	39.8 (2.6)	0.92 (0.66,1.27)	42.5 (2.9)	0.61 (0.42,0.90)
Q3	36.7 (2.7)	0.32 (0.23,0.46)	30.6 (2.7)	0.46 (0.32,0.64)	44.7 (2.9)	0.89 (0.64,1.25)	44.8 (2.6)	1.02 (0.76,1.37)	29.7 (2.8)	0.40 (0.28,0.59)
Q4	30.7 (2.7)	0.32 (0.22,0.44)	23.9 (2.5)	0.17 (0.12,0.24)	44.4 (2.5)	0.90 (0.66,1.22)	42.1 (2.9)	0.97 (0.71,1.34)	18.9 (2.1)	0.14 (0.10,0.20)
p for trend	<.0001	<.0001	<.0001	<.0001	0.076	0.5331	0.8551	0.9641	<.001	<.0001

*MHO was defined as a person who satisfies 2 or fewer criteria of metabolic syndrome. Categorical variables %(SE), analyzed by CHI-Square test.

Q1, Q2, Q3, and Q4 mean first to fourth quartile respectively. Adjusted by age, smoking, drinking, and physical activity.

BMI, body mass index (kg/m^2^); WC waist circumference (cm); ASM, weight adjusted appendicular skeletal mass; FAT total body fat (%); WHtR, waist to height ratio.

### Prevalence of MHO, according to different definitions and ages

**Figure 1:**
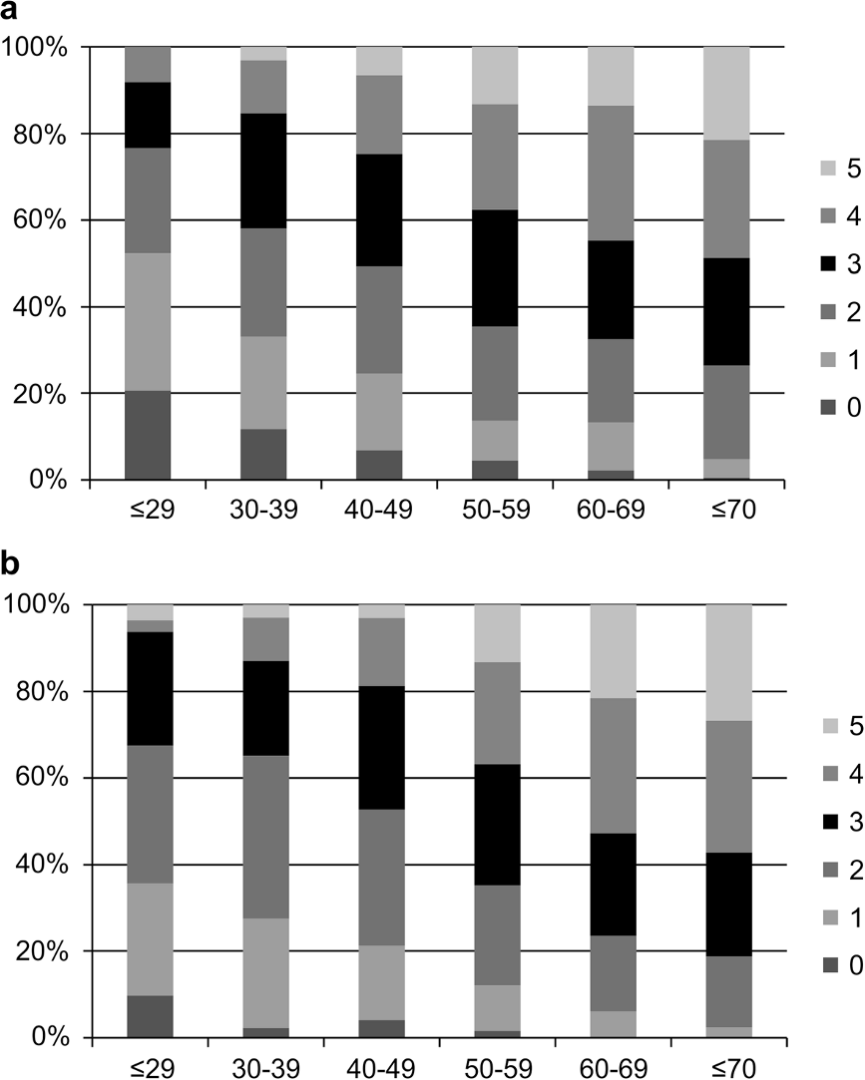
**Proportion of obese patients who have metabolically healthy obesity (MHO) according to age groups with different numbers (0–5) of MS risks. a.** The MHC proportion tends to decrease as age increases. This association remains for each considered definition of MHO. **b.** MHO, metabolically healthy but obese; MS, metabolic syndrome. *Data are expressed as %.

As presented in Figure 1, the proportion of MS subject increased with age among both men and women (*p* trend < 0.0001). The prevalence of MHO strongly depended on its exact definition. Indeed, if the diagnosis of MHO excluded individuals who met 2 or more criteria of MS, then 24.5% of male aged 40–49 years would have MHO. In contrast, if the diagnosis of MHO excluded individuals who met any criteria of MS, only 6.8% of men aged 40–49 years would have MHO.

### Accuracy of MHO diagnosis using obesity indicators

**Figure 2:**
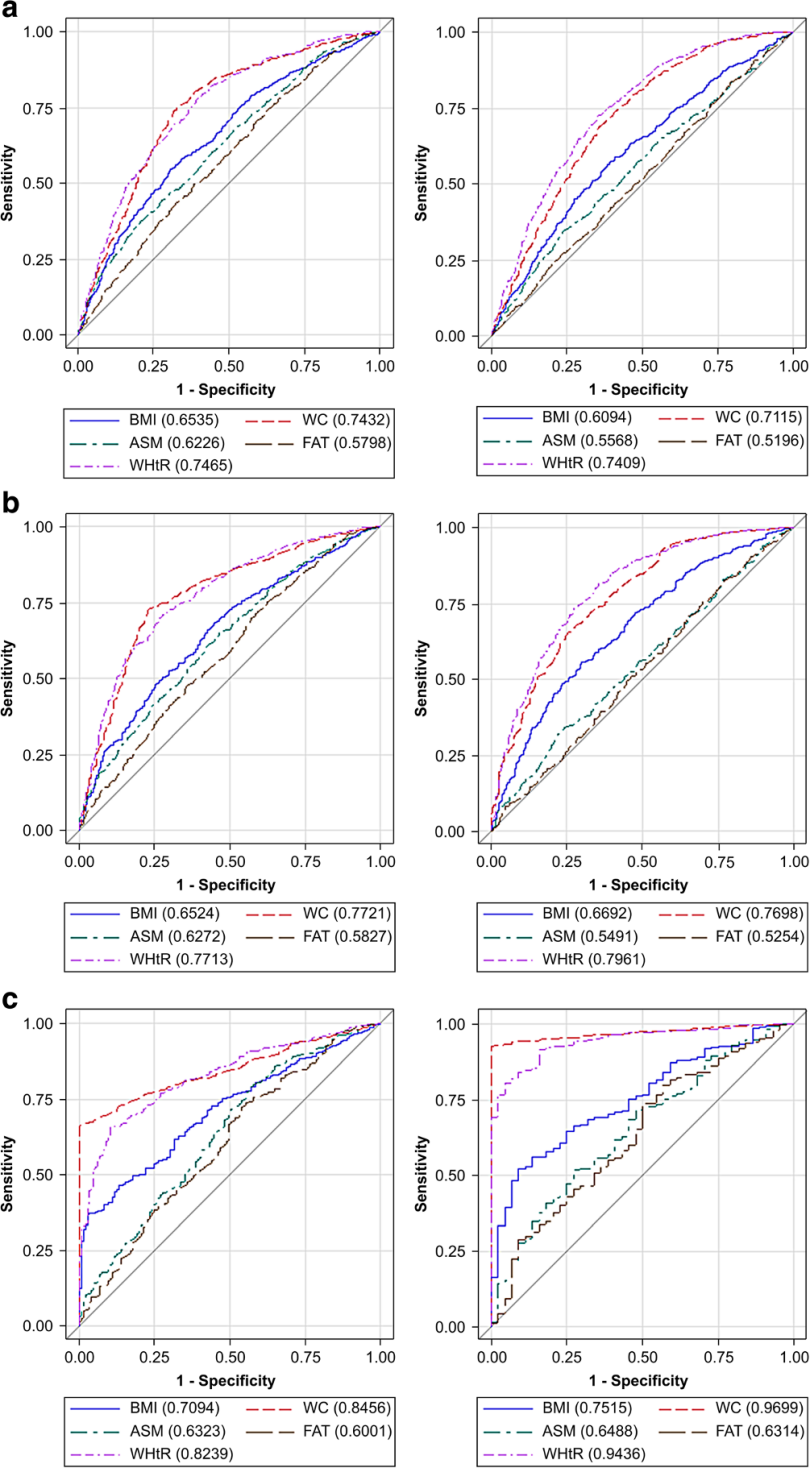
**Receiver operating characteristic (ROC) curves for obesity indices. a.** Metabolically healthy obesity (MHO) criteria ≤2, **b.** MHO criteria ≤1, **c.** MHO criteria = 0. The blue lines indicate body mass index (BMI), the green lines indicate appendicular skeletal muscle (ASM), the red lines indicate waist circumference (WC), the purple lines indicate waist to height ratio (WHtR), the brown lines indicate body fat % (FAT), and the red lines are reference lines. The obesity indices showed different sensitivities and specificities. WC and WHtR were the most sensitive indicators of MHO, as defined by the fulfillment of MS risk factors = 0 (AUC = 0.846 for WC and 0.824 for WHtR in men, and 0.970 for WC and 0.944 for WHtR in women).

AUCs were calculated to investigate the diagnostic accuracy of obesity indicators for MHO (Table 2 and Figure 2). When MHO was defined by ≤2 criteria of MS, waist circumference and WHtR had relatively high diagnostic accuracies for MHO (AUC = 0.7432 and 0.7465 for men, and AUC = 0.7115 and 0.7409 for women, respectively). When MHO was defined by the absence of any criteria of MS, waist circumference and WHtR had even higher diagnostic accuracies among both men (AUC = 0.8456 and 0.8239, respectively) and women (AUC = 0.9699 and 0.9436, respectively). Waist circumference and WHtR were consistently accurate indicators of MHO.

## Discussion

Obesity is strongly associated with CVD and diabetes, but a study has demonstrated that obesity is not independently associated with either increased overall mortality or, more specifically, increased CVD mortality [9]. Meigs et al.’s findings suggest that the metabolic state of obese persons is more closely related to disease development than is obesity itself. Compared with other forms of obesity, MHO is metabolically closer to the normal health state and is associated with a lower risk for CVD and diabetes. Various studies have reported that the incidence of MHO differs according to race. In a previous study on Korean adults, the prevalence of MHO was 49.0% [10], which is similar to the 43.2% observed in our study.

Currently, there is no consensus regarding the criteria for diagnosing MHO. In several previous studies, MHO incidence has been assessed based on subsets of the 5 criteria for diagnosing metabolic syndrome, as we have done here [6, 17–20]. Using the same definitions of MHO as previous reports, we observed that the prevalence of MHO decreased with age among obese persons; this result agrees with previous reports [8]. The decreasing age-specific prevalence of MHO is believed to be a result of decreasing insulin resistance and muscle mass at older ages [21].

Although lifestyle changes and weight loss are commonly recommended to individuals with other types of obesity, it is unclear whether they should be recommended to individuals with MHO. One study showed that weight reduction in MHO subjects did not lead to much metabolic improvement, but was associated with improvements in selected cardio-metabolic risk factors [22]. It is important to remember that even subjects with MHO have some dysmetabolic features, such as higher insulin, insulin resistance, non-HDL cholesterol and C-reactive protein levels than individuals with normal weight, as well as lower high-density lipoprotein cholesterol (HDL) levels than individuals with normal weight. Therefore, individuals with MHO also need diet and lifestyle interventions [23].

At present, BMI is widely used as a criterion for obesity, although it provides unsatisfactory sufficient assessments of metabolic or health state. Recently, Hung et al. demonstrated that BMI reflects visceral fat more accurately than does body adiposity index [24]. However, in this study, we observed that BMI was less accurate than waist circumference or WHtR in determining MHO. In addition, although body adiposity index has been shown to provide a good assessment of body fat percentage [25], we did not observe this for our results. Our results show that the female MHO group in our study had body weights and fat percentages similar to those of the female MS group. Further, body fat percentage was a less accurate indicator of metabolic state in obese patients than was waist circumference or WHtR. It is not surprising that WC and WHtR predict MHO better than the other obesity indicators because one of the criteria for MS is WC, and MHO is defined based on the major criteria of MS. However, we believe that WC and WHtR offer better predictive performance because the distribution pattern of excess fat is more closely related to CVD risk than total fat.

Waist circumference was considered a better measure of visceral fat than waist to hip ratio [26]. In this study, we used WHtR rather than waist to hip ratio because hip circumference was not available. When MHO was defined as ≤ 2 criteria of MS, both WHtR and waist circumference were accurate. However, it has also been reported that anthropometric measurements (including waist circumference and WHtR) are not suitable markers of deteriorated lipid profiles in severe obesity (BMI > 40 kg/m^2^) [27]. Although we attempted to analyze the subgroup of subjects with BMI > 35 kg/m^2^, we could not find similar results because of small number of subjects (n = 52).

WHtR is known to be a risk factor for arteriosclerosis [28], and has been reported to be a better predictor of CVD than waist circumference, especially in men [29]. However, in our study, waist circumference was not inferior to WHtR as a predictor of MS, including after stratifying by gender and for most levels of metabolic risks.

In clinical practice, MS is diagnosed on the basis of body measurements such as height, weight, waist circumference, and blood pressure and laboratory tests such as lipid and blood sugar. However, our results indicated that even simple body measurements can be used to accurately predict the metabolic state of obese patients. This may be advantageous because body measurements are faster and less costly.

In this study, insulin sensitivity was not a criterion for diagnosing MHO, which is a potential limitation of our research; however, we attempted to minimize this error by including fasting blood sugar and the use of diabetes medication. Our findings are limited to the Asian population and should be confirmed in other ethnic groups and regions. However, the KNHANES study sample that we used was large and nationally representative, including several years of data and numerous covariates.

## Conclusions

The results of this study demonstrate that waist circumference and WHtR can serve as important indicators of metabolic status for persons who have been classified as obese based on their BMIs. Additional research is needed on obesity indicators for MHO. It is essential to obtain a consensus definition of MHO, establish diagnosis criteria, and provide additional methods for distinguishing MHO from other forms of obesity.

## Authors’ information

YKR is a professor of College of Medicine, Hallym university and a supervisor of the department of Family Medicine of Kangnam Sacred Heart Hospital; a board member in the Korean Academy of Family Medicine and the Korean Geriatrics Society.

MKC is an assistant professor in the College of Medicine, Hallym University and a medical doctor in the Department of Family Medicine, Kangnam Sacred Heart Hospital, YAH is a medical doctor in a local clinic.
